# Simulation of thoracic endovascular aortic repair in a perfused patient-specific model of type B aortic dissection

**DOI:** 10.1007/s11548-024-03190-3

**Published:** 2024-06-07

**Authors:** Lukas Mohl, Roger Karl, Matthias N. Hagedorn, Armin Runz, Stephan Skornitzke, Malte Toelle, C. Soeren Bergt, Johannes Hatzl, Christian Uhl, Dittmar Böckler, Katrin Meisenbacher, Sandy Engelhardt

**Affiliations:** 1 Department of Cardiac Surgery, Heidelberg University Hospital, Heidelberg, Germany; 2 Department of Internal Medicine III, Department of Cardiology, Heidelberg University Hospital, Im Neuenheimer Feld 410, 69120 Heidelberg, Germany; 3 German Centre for Cardiovascular Research (DZHK), Partner Site Heidelberg/Mannheim, Heidelberg, Germany; 4 DKFZ, German Cancer Research Center, Heidelberg, Germany; 5 Department of Vascular Surgery and Endovascular Surgery, University Hospital Heidelberg, Heidelberg, Germany; 6 Diagnostic and Interventional Radiology, Heidelberg University Hospital, Heidelberg, Germany; 7 Department of Vascular Surgery, RWTH Aachen, Aachen, Germany

**Keywords:** Aortic dissection, Patient-specific aorta, Hemodynamic aortic flow loop, TEVAR simulation, In vitro phantom, 3d printing

## Abstract

**Purpose:**

Complicated type B Aortic dissection is a severe aortic pathology that requires treatment through thoracic endovascular aortic repair (TEVAR). During TEVAR a stentgraft is deployed in the aortic lumen in order to restore blood flow. Due to the complicated pathology including an entry, a resulting dissection wall with potentially several re-entries, replicating this structure artificially has proven to be challenging thus far.

**Methods:**

We developed a 3d printed, patient-specific and perfused aortic dissection phantom with a flexible dissection flap and all major branching vessels. The model was segmented from CTA images and fabricated out of a flexible material to mimic aortic wall tissue. It was placed in a pulsatile hemodynamic flow loop. Hemodynamics were investigated through pressure and flow measurements and doppler ultrasound imaging. Surgeons performed a TEVAR intervention including stentgraft deployment under fluoroscopic guidance.

**Results:**

The flexible aortic dissection phantom was successfully incorporated in the hemodynamic flow loop, a systolic pressure of 112 mmHg and physiological flow of 4.05 L per minute was reached. Flow velocities were higher in true lumen with a up to 35.7 cm/s compared to the false lumen with a maximum of 13.3 cm/s, chaotic flow patterns were observed on main entry and reentry sights. A TEVAR procedure was successfully performed under fluoroscopy. The position of the stentgraft was confirmed using CTA imaging.

**Conclusions:**

This perfused in-vitro phantom allows for detailed investigation of the complex inner hemodynamics of aortic dissections on a patient-specific level and enables the simulation of TEVAR procedures in a real endovascular operating environment. Therefore, it could provide a dynamic platform for future surgical training and research.

## Introduction

**Fig. 1:**
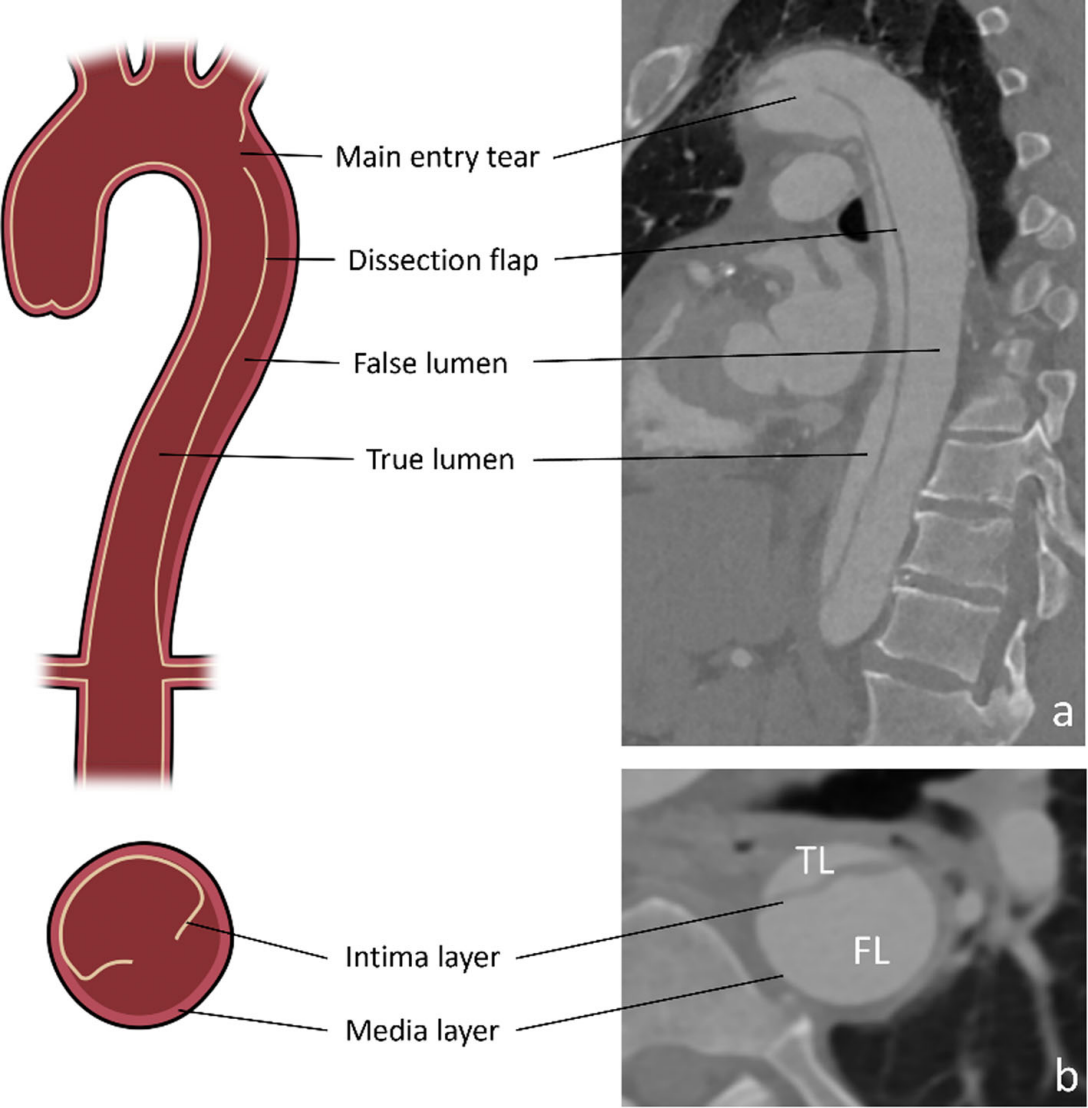
Anatomical features of type B Aortic dissections in illustration and CTA imaging (**a** and **b**) TL: True Lumen FL: False Lumen

Acute aortic dissection is a rare but life-threatening disease associated with a high mortality. Affecting between 2.6 and 3.5 in 100.000 inhabitants/year, it is one of the most common causes for emergency aortic surgery [1]. Aortic dissections are characterized by a tear in the intima layer (main entry) of the aortic wall, causing bleeding within the aortic wall layers, thus dividing the wall integrity into two lumina: a true and a false lumen (Fig. 1). Both lumina are separated by the detached intima layer which is referred to as a dissection flap. The dissection is classified based on the location of the main entry [2]. Stanford type A Dissections are characterized by a main entry in the ascending aorta or the aortic arch, while Stanford type B aortic dissections (TBAD) feature a main entry distal to the orifice of the left subclavian artery. In comparison to type A dissections which require emergent open surgery, uncomplicated TBADs are usually treated using medication (“best medical treatment”). Complicated TBADs however, characterized by signs of downstream malperfusion, uncontrollable hypertension or pain, aortic expansion or aortic rupture lead to a worse outcome. These cases usually require thoracic endovascular aortic repair (TEVAR) [3]. In this procedure, an endovascular stentgraft is inserted via the femoral artery and deployed inside the true lumen of the aorta. A stentgraft is a tube composed of fabric (Dacron or PTFE) supported by a metal mesh called a stent. Its purpose is to seal the main entry and redirect blood flow into the true lumen. TEVAR aims at stabilizing the aorta and initiate thrombosis of the false lumen therefore preventing aortic rupture and improving distal organ perfusion [4].

However, steering the material into the true lumen and subsequently placing the stentgraft correctly is not an easy endeavor. The different lumina are sometimes difficult to distinguish and a misplaced stentgraft might end up fatal. Treating aortic dissections, including TEVAR procedures, require high expertise and years of experience [5]. Especially for young, unexperienced vascular surgeons, endovascular interventions are associated with a steep learning curve and need regular interventions to reach consistent quality [6]. Due to the low case number of complicated TBADs there is a demand for better TEVAR training platforms for vascular surgeons.

*In-vitro* aortic phantoms have been a valuable tool to investigate the hemodynamics of aortic pathologies. For abdominal aortic aneurysms they have already been used to simulate treatment procedures [7–9]. For TBADs, the development of *in-vitro* phantoms is much more challenging from a technical point of view due to the complex and fragile anatomy of the dissection flap that creates an additional potentially helical lumen. In particular, TBADs regularly extend all the way down to the iliac bifurcation, requiring a full-size aortic model with all relevant branching vessels. Therefore, only few in-vitro phantoms have been ever developed for TBADs. Phantoms that replicate patient-specific anatomy and pathology are challenging to manufacture but offer unique insights into the hemodynamics and treatment challenges of individuals. Morris et al. [10] and Chen et al. [11] developed full-size aortic dissection models with patient-specific anatomy using a complex multistep wax casting process or self-developed silicone application machines. However, easy and highly customizable manufacturing are important factors in aortic phantom development; therefore, we propose the use of Polyjet 3d printing for aortic phantom production. Polyjet 3d printing is well established and commercially available technology offering flexible materials and repeatable results. Zimmermann et al. [12] have experimented with 3d printing for their dissection models, but their phantom did not incorporate a full-size aorta or branching vessels.

Treatment of complicated TBAD requires the deployment of a stentgraft inside the aorta. Birjiniuk et al. [13] deployed stentgrafts inside an aortic dissection model although their models were idealized phantoms without branching vessels and not patient-specific. To the best of our knowledge, performing TEVAR and deploying a stentgraft inside a perfused individualized TBAD model remains a novelty. This could potentially enable patient-specific surgical training and pre-procedural planning for individual cases. Table 1 provides an overview of existing approaches.

**Table 1:** Comparison of existing methods for 3d printing of aortic dissection models

	Patient-specific	Entire aorta/all branching vessels	Production technique	Flexible material	Perfusion	Intervention	Tested compatibility with imaging
Morris et al. [10]	Yes	No	Silicone molding	Silicone	Yes	No	US
Chen et al. [11]	Yes	Yes	Silicone molding	Silicone	Yes	No	US
Zimmermann et al. [12]	Yes	No	Polyjet 3D Printing	Agilus30	Yes	No	MRI
Birjuniuk et al. [13]	No	No	Silicone molding	Silicone	Yes	Yes	MRI
Mohl et al. (ours)	Yes	Yes	Polyjet 3D-Printing	TangoPlus	Yes (pulsatile)	TEVAR	CTA, Fluoroscopy, US

The main goals of this study were three-fold. (1) First, to develop and validate a full-size patient-specific aortic dissection phantom perfused by a hemodynamic flow loop. (2) The system should enable TEVAR simulation and stentgraft placement inside the phantom in a realistic operating environment. (3) The model should be compatible with common diagnostic and surgical imaging modalities of computed tomography angiography (CTA), fluoroscopy and ultrasound to investigate TBAD hemodynamics and enable guidance during stentgraft placement.

## Materials and methods

### Patient-specific aortic phantom

#### Virtual model

**Fig. 2:**
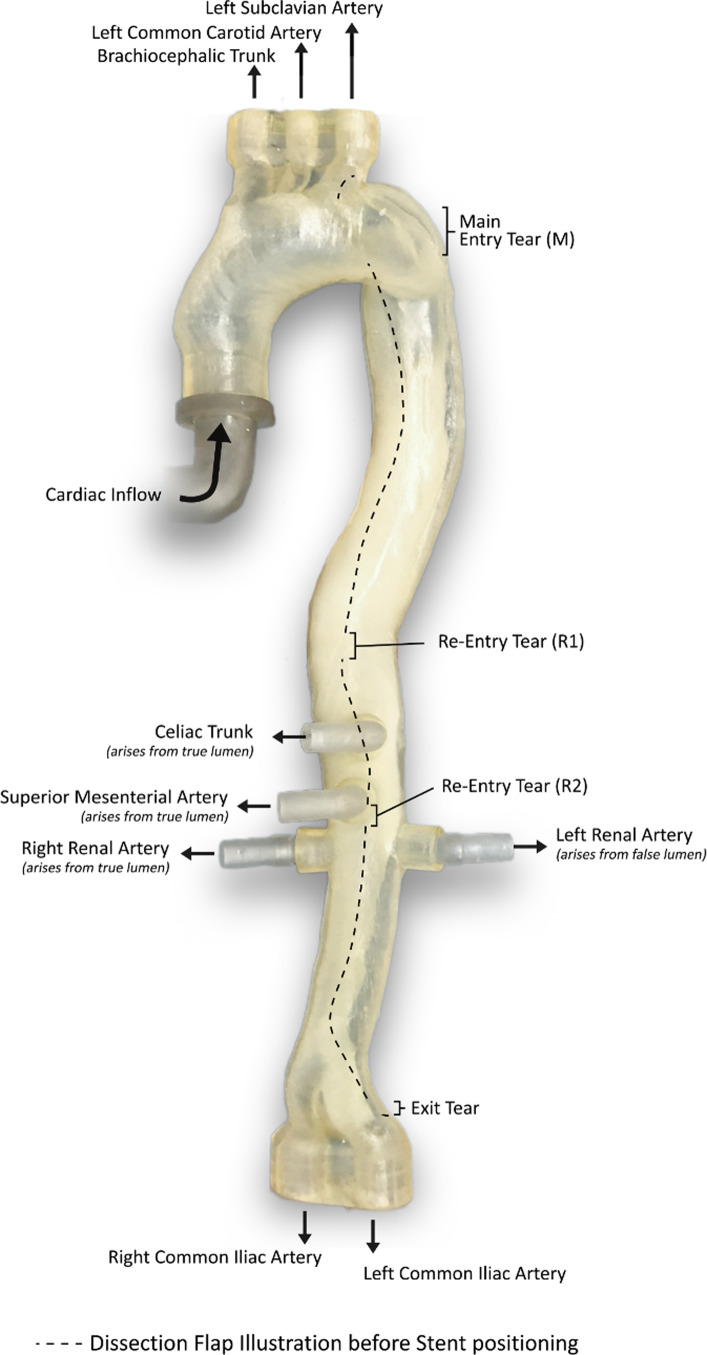
Phantom schematic illustrating aortic branching vessels, main entry (M), Reentries (R1,R2) and Dissection flap

A computed tomography angiography (CTA) dataset in Digital Imaging and Communications in Medicine (DICOM) format of a patient with complicated TBAD was selected by vascular surgeons of Heidelberg University Hospital. The scan was acquired using a standardized institutional CTA protocol with 1 mm slice thickness at 60% of the R-R interval correlating to late diastole. [14] Patient consent and ethical approval was obtained from the Ethics Committee of the Faculty of Medicine of Ruprecht-Karls-University Heidelberg, Germany (S-158/2015). The scan featured one main entry close to the orifice of the left subclavian artery in the descending part of the aortic arch. Furthermore, two reentries were identified at different heights along the dissection flap (Fig. 2).

**Fig. 3:**
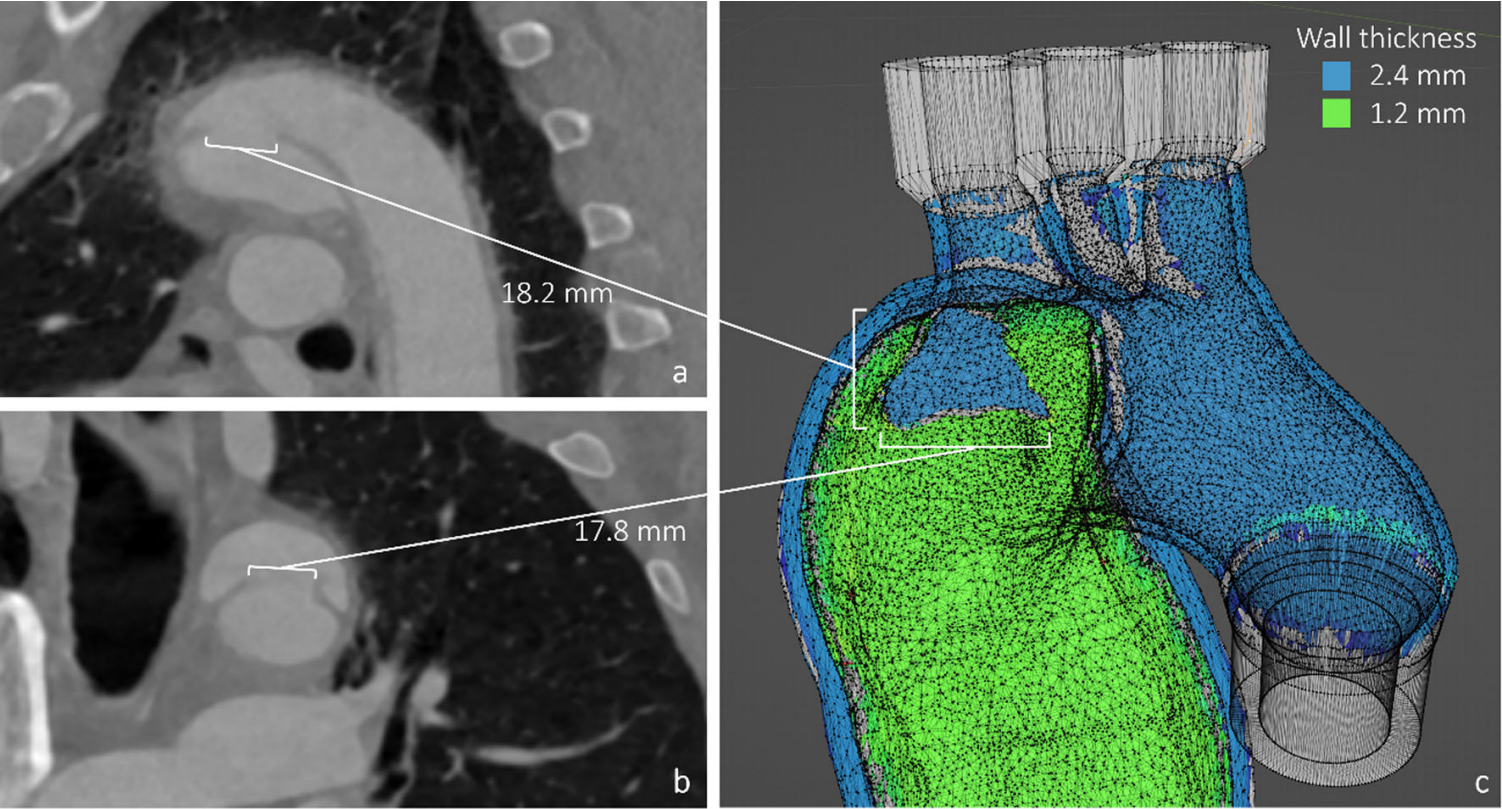
Main entry sight in sagittal view (**a**), coronal view (**b**) and 3d geometry (**c**)

The Medical Imaging Interaction Toolkit (MITK) software was used to manually segment the dataset including main aortic branching vessels (brachiocephalic trunk, left common carotid artery, left subclavian artery, celiac trunk, superior mesenteric artery, renal arteries, common iliac arteries). The main entry and the two reentries were identified by vascular surgeons and incorporated into the model with their anatomic shape and diameter (Fig. 3).

**Fig. 4:**
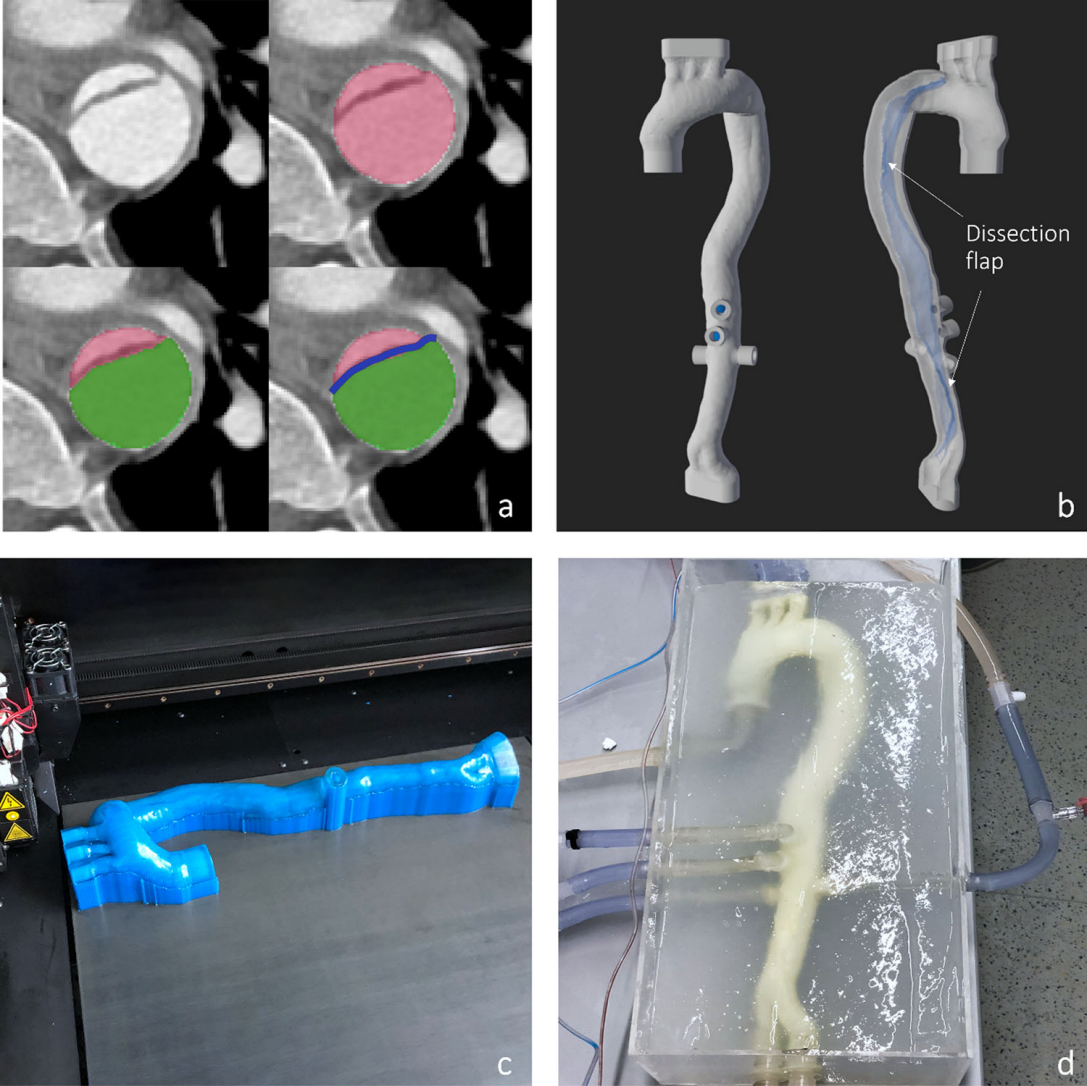
Phantom manufacturing process: CTA data segmentation. Whole lumen [red], false lumen [green], dissection flap [blue] (**a**), 3d model merging and clean up (**b**), printing and support material removal (**c**), finished phantom (**d**)

The different segmentations were converted to 3d geometry and imported in the open-source software Blender Version 3.0 (Blender Foundation, Amsterdam, Netherlands) for post processing and printing preparations: To generate the outer aortic wall a segmentation of the whole lumen, combining both true and false lumen of the aorta, was used. Extruding this shape using a solidify modifier, the outer aortic wall with a uniform thickness of 2.4 mm was created. The dissection flap was formed using a second segmentation of only the false lumen and then removing all the faces that intersected with the outer wall segmentation using Blenders shrink-wrap and Boolean modifiers. The remaining 2-dimentional flap geometry was extruded by 1.2 mm in the direction of the true lumen to form the 3d geometry of the dissection flap with a uniform thickness (Fig. 4a). Before combining the flap with the aortic wall through a Boolean operation, the flaps intersection position with the wall were altered giving the flap more leeway to move and get expanded by a stentgraft (Fig. 4b). This way, the original location of the flap is preserved, while allowing for substantial true lumen stentgraft dilatation as observed in vivo CTA data after interventions [15], that would otherwise not be possible due to limitations of the 3d printing material. Afterward, the segmented model was converted to a smooth polygonal representation. On all branching vessels connectors were modeled using computer aided design to ensure a watertight seal with the connecting tubes.

#### 3d printed aortic phantom

The finished patient-specific aortic model was printed on an industrial Stratasys Objet-500 Connex 3 3d printer (Fig. 4c) with TangoPlus, a flexible and translucent resin (both Stratasys Inc., Rechovot, Israel) and SUP706B as support material. TangoPlus was chosen since it closely matches the properties of the outer aortic wall [16]. After manually removing as much support material as possible the phantom was soaked in an alkaline bath to dissolve the remaining support material inside the phantom. After printing we thoroughly evaluated the model for defects. If small leaks were observed due to printing imperfections, they were repaired by manually injecting a tiny amount of printing resin into the crack and curing it with a UV flashlight. If ruptures occurred in sensitive positions, for example not fixable spots at the dissection membrane, we had to reprint the model. An acryl box was built for the phantom which got embedded in 2% Laponite-Gel (BYK-Chemie GmbH, Wessel, Germany) to mimic the extravascular matrix and facilitate good ultrasound visibility.

#### Hemodynamic flow loop

**Fig. 5:**
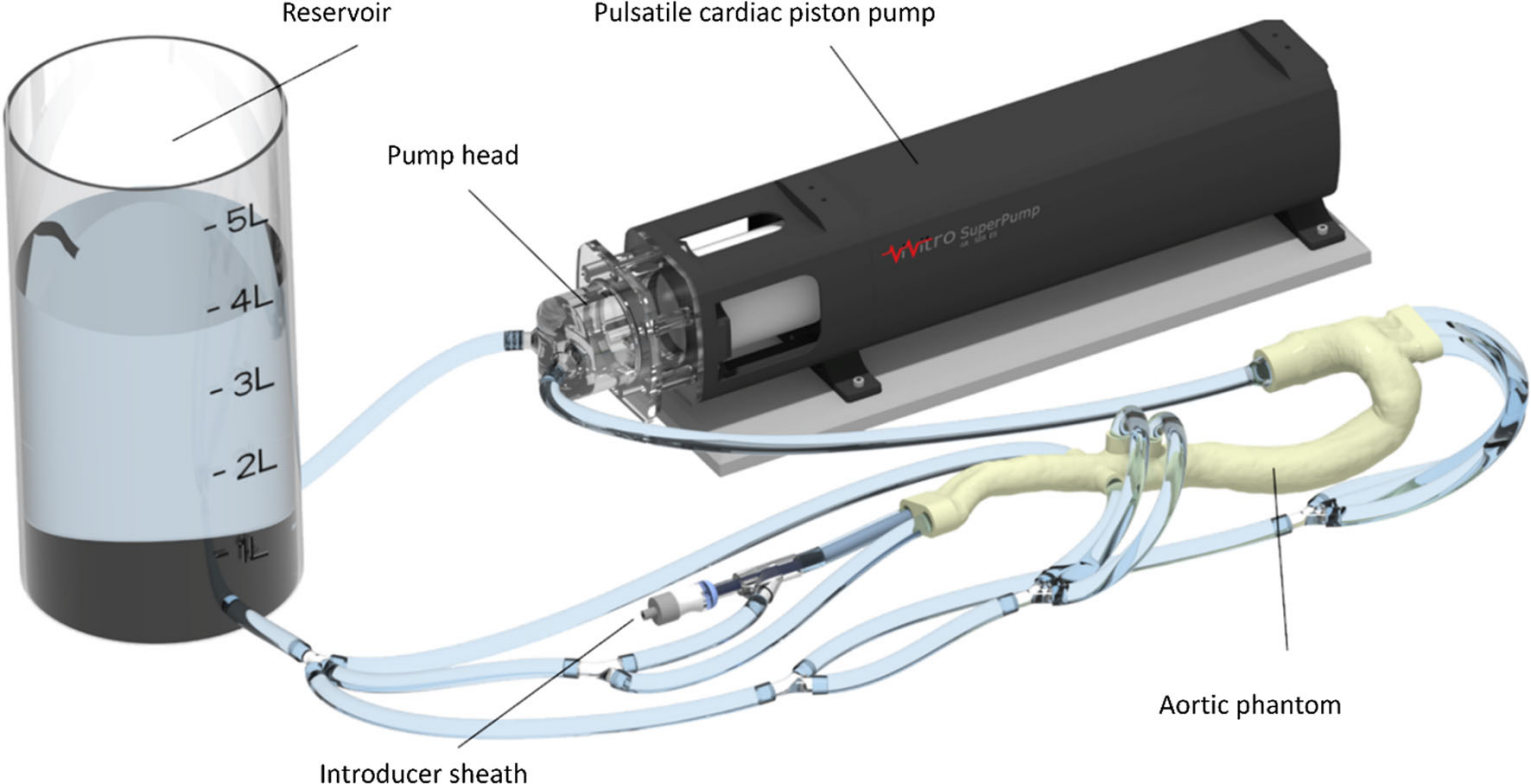
Aortic flow loop. A cardiac piston pump induces hemodynamic flow through the aorta. Each branching vessel is connected to the reservoir. An introducer sheath within the right iliac artery enables stent-grafting such as thoracic endovascular aortic repair (TEVAR)

The phantom was then included in a hemodynamic flow loop (Fig. 5). Each branching vessel had a separate outflow to enable individual flow and pressure measurements. A realistic pulsatile flow through the aortic phantom is provided by a cardiac piston pump (Superpump, Vivitro Labs Inc., Victoria, Canada), also utilized, e.g., by Karl et al. [17]. A fluid mixture of 70% distilled water and 30% glycerol was used as blood mimicking fluid (BMF) to achieve a viscosity that closely resembles human blood [18]. Flow and pressure values were measured by ultrasound Sonoflow CO.55 V2.0 sensors (SONOTEC GmbH, Halle (Saale), Germany) and HONEYWELL ABPDRRT005PG2A5 sensors (Honeywell International Inc., North Carolina, USA), respectively. Femoral access for catheter devices was enabled by including a 24 Fr Gore DrySeal Flex Introducer sheath (W. L. Gore & Associates, Inc. Delaware USA) into the right iliac artery.

## Experiments

During all experiments the cardiac piston pump was set to a frequency of 80 bpm with a stroke volume of 70 ml to mimic physiological conditions.

### Ultrasound and flow

**Fig. 6:**
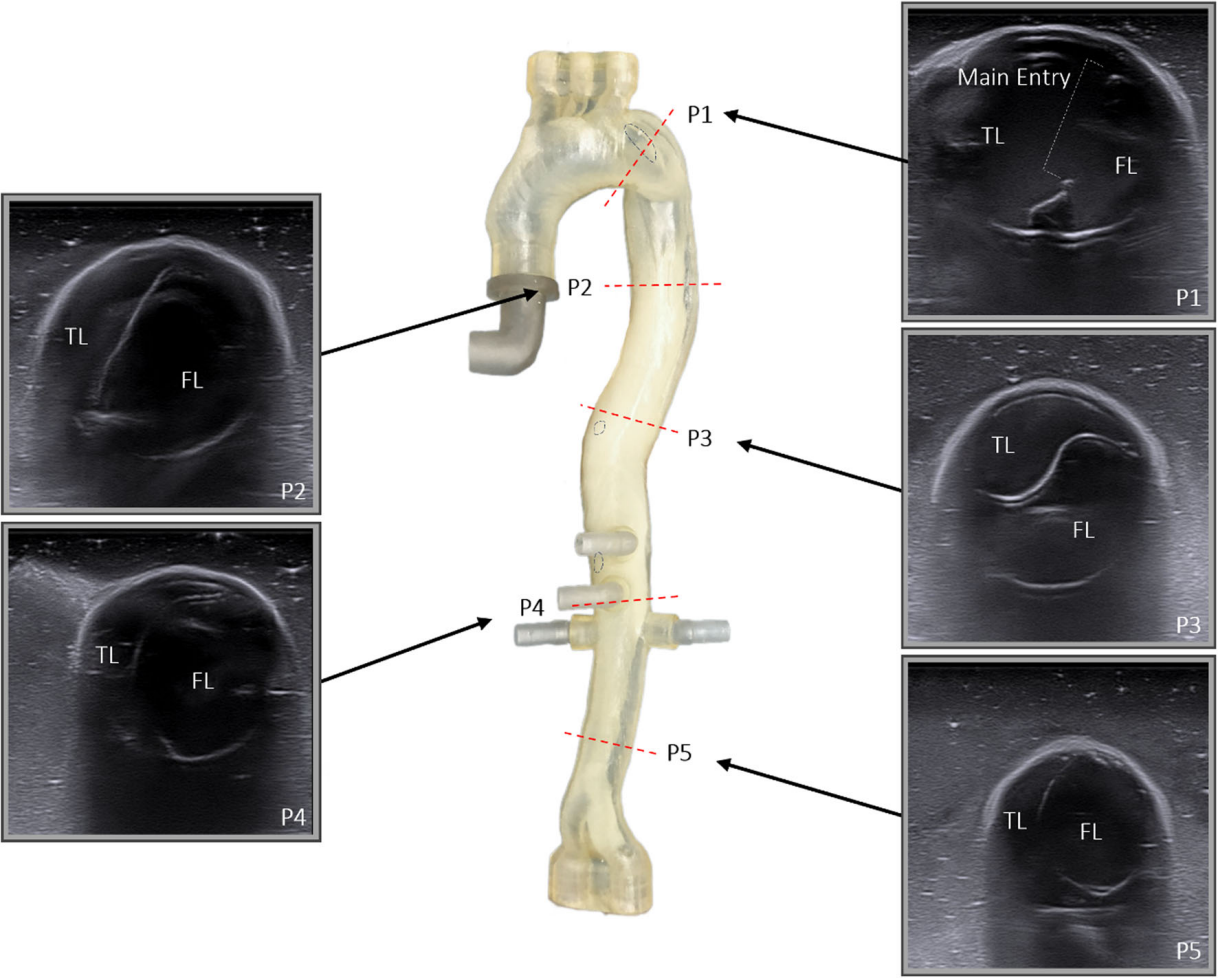
Ultrasound axial views with true lumen and (TL) false lumen (FL) divided by dissection flap

**Fig. 7:**
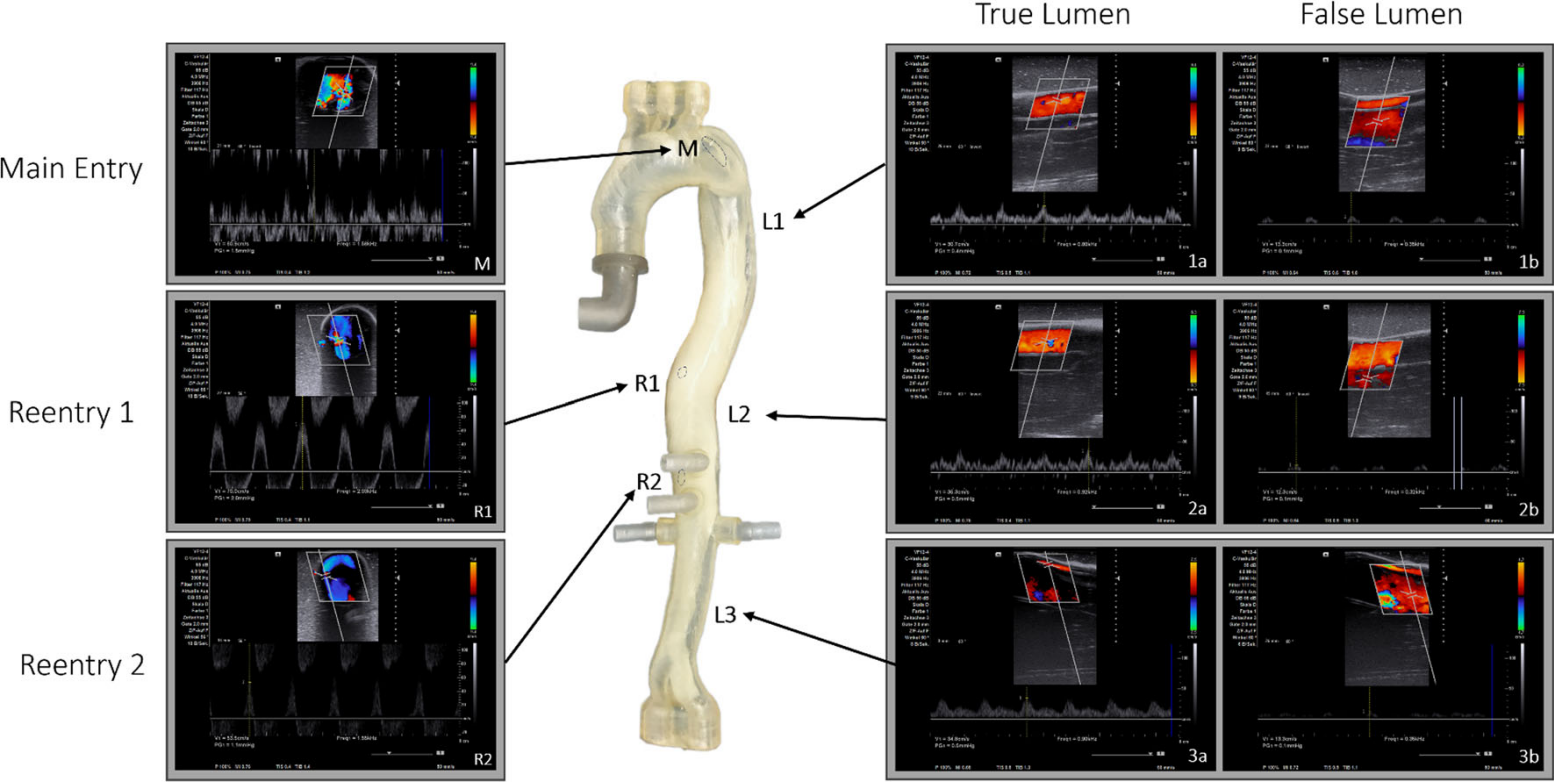
Ultrasound Velocity in true lumen (**a**) false lumen (**b**), main entry (M) and reentries (R1,R2)

Ultrasound images of the phantom were acquired before TEVAR intervention by an Acuson X700 Ultrasound Machine in combination with a VF12-4 probe (both Siemens Healthcare GmbH, Erlangen, Germany) on five axial (Fig. 6) and three longitudinal (Fig. 7) previously defined positions along the dissection to investigate flap movement and fluid velocities using doppler imaging. This was done separately for true lumen, false lumen and at the sights of the main entry and the two reentries. To meet the backscatter properties of blood 1% corn starch was added to the BMF [18]. Furthermore, flow and pressure measurements were obtained for 30 s at each aortic branching vessel.

#### Computed tomography angiography

To evaluate TEVAR performance in terms of stentgraft placement and dissection membrane course, CTA scans were performed prior and after TEVAR by using a Somatom Force CT scanner (Siemens Healthcare GmbH, Erlangen, Germany). 0.67% ACCUPAQUE™ 300 contrast agent (GE Healthcare Technologies, Chicago, Illinois, USA) was added to the BMF to reach a mixture of 6 mg/ml. CTA scans were obtained under flow to ensure an equal distribution of contrast agent.

#### Digital subtraction angiography and thoracic endovascular repair

After visual assessment of the CTA images the flow loop was placed in a hybrid operating room to mimic the workflow of real TEVAR. The aortic phantom was aligned to the robotic imaging system (Artis Pheno by Siemens Healthineers, Forchheim, Germany) to enable fluoroscopy. Using the introducer sheath in the right iliac artery, a soft wire with a pigtail catheter was introduced into the aortic phantom. The catheter was navigated under fluoroscopy. During advancement of the catheter, contrast agent was applied manually to increase the distinction of the true and false lumen and improve the navigational guidance.

Next, the stentgraft device (E-vita thoracic 3G, 230 mm length / 33 mm diameter, ARTIVION GmbH, Hechingen, Germany) was inserted. After positioning the stentgraft at its landing zone fluoroscopy was obtained again to ensure proper positioning. Next, the stentgraft was meticulously deployed and fluoroscopy was performed to assess proper sealing of the main entry.

## Results

A patient-specific aortic flow loop was developed and validated to be used for simulation of TEVAR and investigation of internal TBAD hemodynamics. Its compatibility with medical imaging technologies of ultrasound, fluoroscopy and CTA were assessed.

### Ultrasound

Through the use of doppler ultrasound, flow velocity and flap movement of the patient-specific TBAD phantom were investigated while connected to the pulsatile flow loop. At all five axial (Fig. 6) and all three longitudinal (Fig. 7) measuring positions, the dissection flap was clearly visible; true and false lumen were easily distinguishable, the main entry and the two reentries could be identified. Radial expansion and relaxation of the aorta were observable with noticeable movement of the dissection flap in axial and longitudinal view.

In longitudinal view flow velocity in the true and false lumen were measured on three different positions along the phantom (Fig. 7). On all three measurement positions true lumen velocity was consistently higher compared to the false lumen. On longitudinal position 1 (L1) the true lumen velocity was 30.7 cm/s, false lumen velocity was 13.3 cm/s. On position 2 (L2) the true lumen velocity was 35.7 cm/s and false lumen velocity peaked at 12.3 cm/s. On the 3rd and lowest position (L3), true lumen velocity was 34.5cm/s and false lumen velocity was 13.3 cm/s (Fig. 7).

Flow through the main entry and the two reentries along the dissection flap was investigated in terms of flow velocity and direction. On the main entry doppler showed a turbulent flow pattern with fluid entering the false lumen from the true lumen with a peak velocity of 60.9 cm/s (M). The much smaller reentries in the abdominal aorta both showed a pulsatile stream of blood with high velocity from the true lumen into the false lumen during the systolic pulse. For reentry 1 (R1) the peak velocity was measured at 70 cm/s, for reentry 2 (R2) at 53,5 cm/s. Noticeably both reentry sights showed a periodically reversed flow from the false to the true lumen in the diastolic phase.

### Flow and pressure

The assessment of flow and pressure levels is crucial for establishing a physiological hemodynamic environment and for studying the impact of TBAD on individual aortic branches.

During systole, the pressure at the aortic root was 112 mmHg, with a flow rate of 4.05 L per minute, resulting in a stroke volume of 50.6 ml. These values, in conjunction with a heart rate of 80 beats per minute, reflect a physiologically appropriate hemodynamic state.

**Fig. 8:**
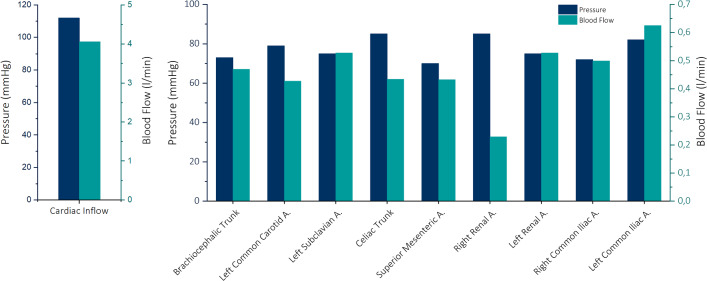
Flow and pressure measured at each aortic branching vessel

Flow and pressure were measured separately on all branching vessels of the aorta. At the brachiocephalic trunk a flow of 0.47 l/min and a pressure of 73 mmHg were measured. The flow through the left common carotid artery was 0.43 l/min with a pressure of 79 mmHg while the left subclavian artery was measured at 0.53 l/min and 75 mmHg of pressure. Further downstream the celiac trunk showed a flow of 0.34 l/min and 85 mmHg of pressure. The flow at the superior mesenteric artery was 0.43 l/min with a pressure of 70 mmHg. The renal arteries were measured at 0.53 l/min and 75 mmHg on the left and 0.23 l/min and 85 mmHg on the right side. The left common iliac artery had a flow of 0.62 l/min and a pressure of 82 mmHg, the right common iliac artery was measured at 0.49 l/min and 72 mmHg of pressure (Fig. 8).

### Computed tomography angiography and TEVAR

**Fig. 9:**
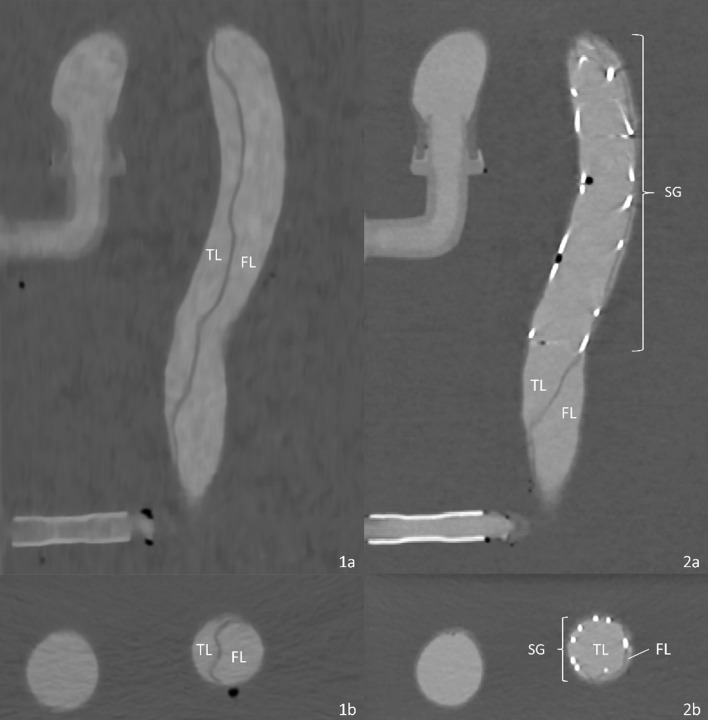
CTA imaging before (1a, b) and after TEVAR (2a, b) TL: True Lumen, FL: False Lumen, SG: Stentgraft

To closely resemble real TEVAR procedures and review the stentgrafts positioning, CTA images of the phantom were acquired before and after the TEVAR intervention (Fig. 9). On pre-procedural CTA images true lumen, false lumen and dissection flap were clearly distinguishable (Fig. 9: 1a, 1b), main entry and reentries could be identified.

**Fig. 10:**
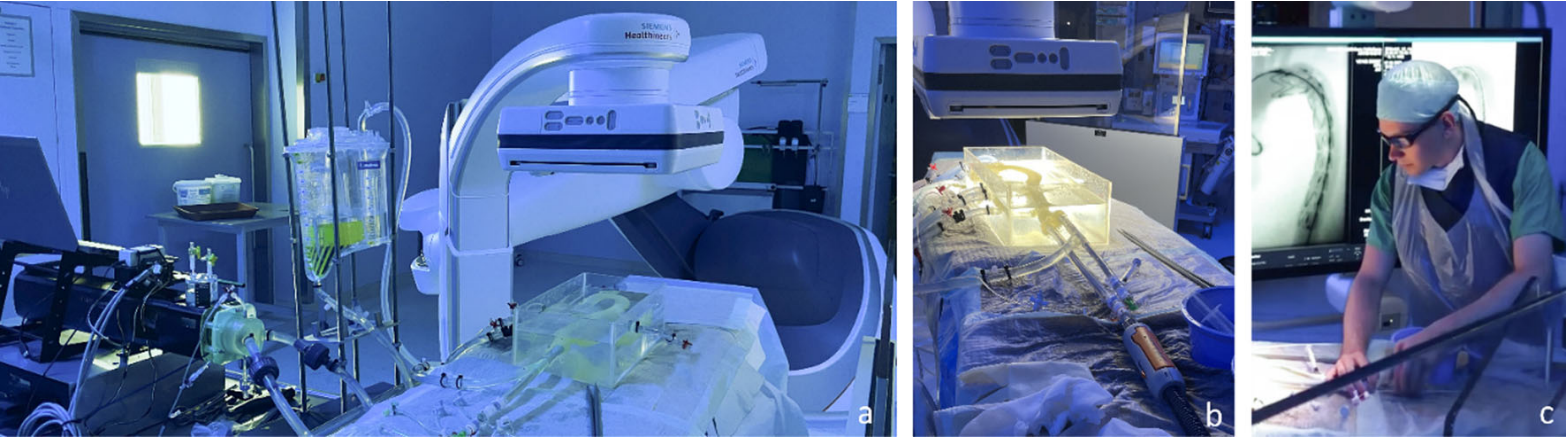
TEVAR procedure in hybrid operating room (**a**), introducer with guiding wire (**b**), stentgraft deployment (**c**)

**Fig. 11:**
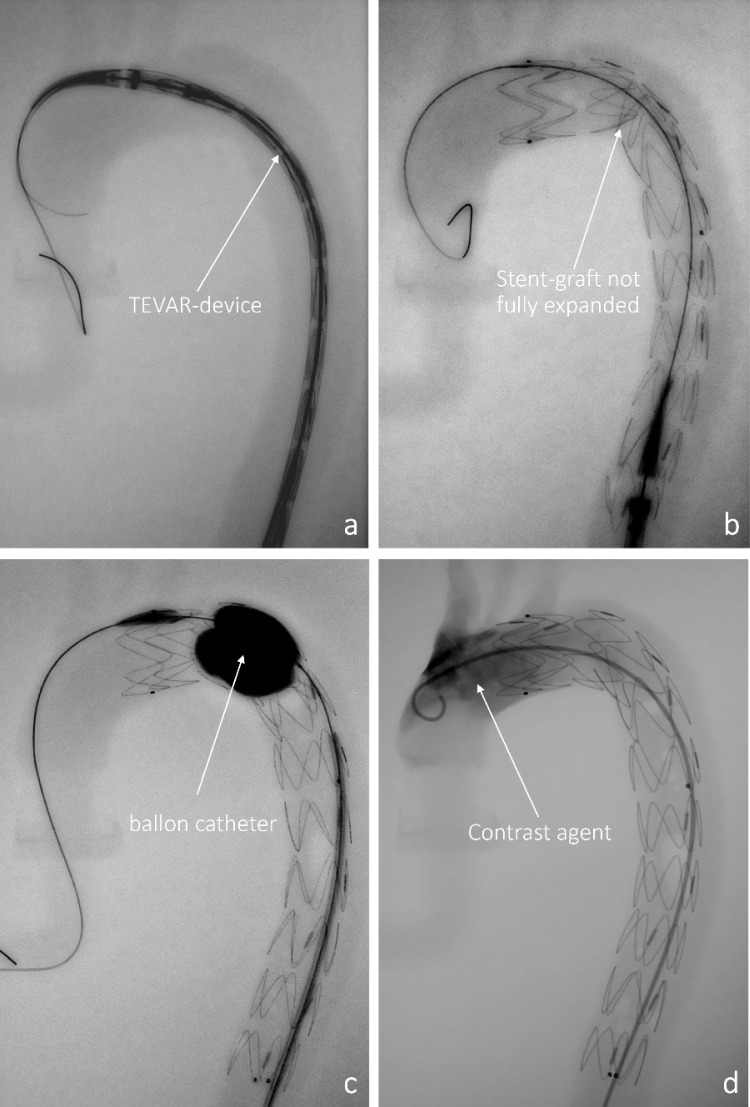
Fluoroscopic imaging during TEVAR procedure before deployment (**a**) not fully extended stent graft (**b**), dilatation with ballon catheter (**c**), checking for endoleaks (**d**)

The TEVAR procedure was performed on the aortic TBAD phantom in a hybrid operating room (Fig. 10). Fluoroscopy confirmed the correct placement of the wire in the true lumen, it further revealed blood flow in the true and false lumen. After advancing the stentgraft over the stiff wire, it was deployed in the desired landing zone covering the orifice of the left subclavian artery, although it was not able to fully expand right away. Using a ballon catheter, the full expansion of the stentgraft was reached without damaging the phantom. (Fig. 11).

**Fig. 12:**
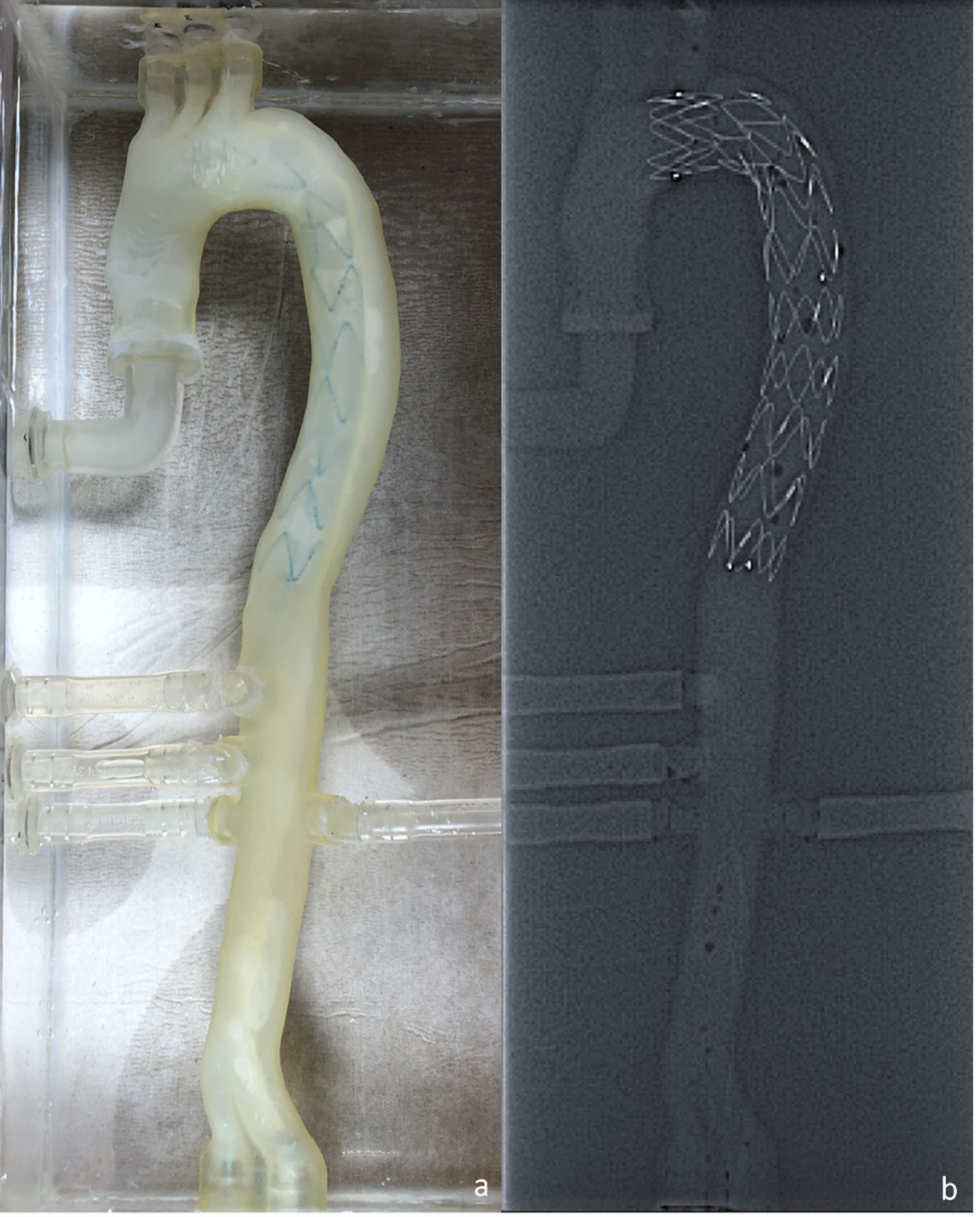
Phantom after TEVAR procedure: external view (**a**) and CT (**b**)

The main entry was fully covered by the stentgraft. Despite that, fluoroscopy revealed a still persistent perfusion of the false lumen through the main entry, referred to as an endoleak type 1a [19], which is a possible complication in TEVAR procedures [20]. Post-interventional CTA Images confirmed the full expansion of the stentgraft (Fig. 12) and its correct placement in the true lumen with consequential dilatation of the true lumen and significant false lumen collapse along the stentgraft (Fig. 92a, 2b).

## Discussion

As far as the authors are aware, this is the first perfused patient-specific 3d printed TBAD phantom that enables TEVAR simulations in a realistic operating environment.

Employing 3d printing to manufacture a flexible full size aortic dissection phantom with dissection flap and all major aortic branching vessels offers multiple benefits over traditional manufacturing methods using silicone molding. Firstly, the used 3d Printer is commercially available and therefore much more accessible potentially enabling a broader use of aortic phantoms for TEVAR simulation. Secondly, 3d printing offers repeatable results and the ability to manufacture exact copies of the used phantom in a short period of time. Thirdly the used Polyjet Printing technology offers the ability to mix different materials while printing. This could potentially be used to alter the material properties of different parts of the phantom, for example altering the stiffness of the dissection flap since it has been reported that its stiffness increases over time in chronical cases [21]. Other patient-specific variants like calcifications could be included in the phantom as well. Compared to solid phantoms of other groups, e.g. [22], a flexible printing material improves the realism of the phantom drastically by mimicking the aortas Windkessel-effect and providing vascular surgeons with more realistic catheter resistance during TEVAR procedure. In this study, the flexible printing material TangoPlus was chosen to print the phantom since it closest matches the material properties of aortic wall tissue [16]. In addition, its translucency enables the observation of catheters and stentgraft deployment from outside for training purposes.

The thickness of the aortic wall and dissection flap were determined experimentally through iterative testing. Setting the outer wall thickness to 2.4 mm produced a reliable model that tolerated the desired fluid pressures consistently while being in the reported range for real aortic wall tissue [23]; the flap was set to 1.2 mm as a result of 3d printing limitations since our tests showed that thinner flaps regularly developed unwanted tears. By using a more durable material for the flap, phantoms with even thinner flaps might be possible. Furthermore, we observed that TangoPlus was prone to crack under chronic stress, so the phantoms had to be stored without tension on the fluid connectors. We also observed that most phantoms would eventually develop leaks after extensive use or longer storage times.

Furthermore, the realism of the phantom is directly influenced by the quality of the CTA scan. Even though we followed an established CTA protocol, it is possible that not all reentries are visible on the preoperative CTA and might only show up during intraoperative fluoroscopy. This is a technical limitation and might limit the realism of the phantom.

Our hemodynamic flow loop can provide a range of physiological blood pressures and heart rates that allow for patient-specific customization and variation. This could particularly be interesting to observe the effects of different blood pressures on TEVAR procedures since some authors suggest lowering the patient’s blood pressure during deployment of the stentgraft [24]. Our flow and pressure measurements on aortic branching vessels illustrate the capabilities of the flow loop to investigate patient-specific hemodynamics and potential organ malperfusion, however, we observed that the course of the tubes have a certain influence on the current measurements, which will be improved in future work.

Ultrasound compatibility allows for investigation of internal TBAD hemodynamics. Doppler ultrasound revealed higher blood velocities in the true lumen compared to the false lumen. These results are in line with the findings of other groups whose in vitro phantoms also showed lower false lumen blood velocities [11, 25]. Furthermore, we found pulsatile flow patterns on main entry and reentry sights which Morris et al. also observed in their silicone phantom.

Due to its compatibility with CTA and fluoroscopy and the implementation of an introducer sheath into the flow loop the patient-specific phantom was successfully tested by vascular surgeons in a hybrid endovascular operating environment performing a TEVAR procedure. While perfused by pulsatile flow different guiding wires were introduced and a stentgraft could be deployed inside the phantom without rupture. Although placed in the desired landing zone the stentgtaft was not able to fully expand without dilation using a ballon catheter. After release of the stentgraft, fluoroscopy revealed a still persistent antegrade perfusion of the false lumen (endoleak type 1a) which is a possible complication of TEVAR, e.g. due to inaccurate deployment [19]. Endoleaks may prevent or delay false lumen thrombosis potentially leading to an ongoing dilatation of the false lumen and therefore might require reintervention [26, 27]. Aortic phantoms could be used to further investigate the underlying reasons for endoleaks or deployment challenges in TEVAR procedures by providing the opportunity to repeat the same intervention while varying TEVAR parameters like stentgraft type, diameter or landing zones and develop strategies that mitigate the risks of endoleaks and its complications. However, due to the high costs of the stentgrafts, research in this area has been limited so far.

## Conclusion

Our hemodynamic flow loop incorporates a flexible patient-specific TBAD phantom. It allows for detailed investigation of TBAD hemodynamics through its compatibility with all relevant medical imaging modalities. A TEVAR procedure was successfully performed on the phantom in a hybrid operating environment and a real stentgraft was deployed. Therefore, this setup is suitable for TEVAR simulation and might enable surgical TEVAR training, research and intervention planning on a patient-specific level.
